# Community Engagement in Cutaneous Leishmaniasis Research in Brazil, Ethiopia, and Sri Lanka: A Decolonial Approach for Global Health

**DOI:** 10.3389/fpubh.2022.823844

**Published:** 2022-02-15

**Authors:** Kay Polidano, Linda Parton, Suneth B. Agampodi, Thilini C. Agampodi, Binega H. Haileselassie, Jayasundara M. G. Lalani, Clarice Mota, Helen P. Price, Steffane Rodrigues, Getachew R. Tafere, Leny A. B. Trad, Zenawi Zerihun, Lisa Dikomitis

**Affiliations:** ^1^School of Medicine, Keele University, Newcastle-under-Lyme, United Kingdom; ^2^Department of Community Medicine, Rajarata University of Sri Lanka, Saliyapura, Sri Lanka; ^3^Department of Social Work, Mekelle University, Mekelle, Ethiopia; ^4^Institute of Collective Health, Federal University of Bahia, Salvador, Brazil; ^5^School of Life Sciences, Keele University, Newcastle-under-Lyme, United Kingdom; ^6^School of Public Health, Mekelle University, Mekelle, Ethiopia; ^7^Department of Psychology, Mekelle University, Mekelle, Ethiopia; ^8^Kent and Medway Medical School, University of Kent and Canterbury Christ Church University, Canterbury, United Kingdom

**Keywords:** qualitative research, ethnography, low-resourced settings, decoloniality, neglected tropical diseases, empowerment, community partnerships, community advisory boards

## Abstract

Cutaneous leishmaniasis (CL) is a parasitic skin disease endemic in at least 88 countries where it presents an urgent, albeit often “neglected” public health problem. In this paper, we discuss our model of decolonial community engagement in the ECLIPSE global health research program, which aims to improve physical and mental health outcomes for people with CL. The ECLIPSE program has four interlinked phases and underpinning each of these phases is sustained and robust community engagement and involvement that guides and informs all activities in ECLIPSE. Our decolonial approach implies that the model for community engagement will be different in Brazil, Ethiopia and Sri Lanka. Indeed, we adopt a critical anthropological approach to engaging with community members and it is precisely this approach we evaluate in this paper. The data and material we draw on were collected through qualitative research methods during community engagement activities. We established 13 Community Advisory Groups (CAGs): in Brazil (*n* = 4), Ethiopia (*n* = 6), and Sri Lanka (*n* = 3). We identified four overarching themes during a thematic analysis of the data set: (1) Establishing community advisory groups, (2) CAG membership and community representation, (3) Culturally appropriate and context-bespoke engagement, and (4) Relationships between researchers and community members. During our first period of ECLIPSE community engagement, we have debunked myths (for instance about communities being “disempowered”), critiqued our own practices (changing approaches in bringing together CAG members) and celebrated successes (notably fruitful online engagement during a challenging COVID-19 pandemic context). Our evaluation revealed a gap between the exemplary community engagement frameworks available in the literature and the messy, everyday reality of working in communities. In the ECLIPSE program, we have translated ideal(istic) principles espoused by such community engagement guidance into the practical realities of “doing engagement” in low-resourced communities. Our community engagement was underpinned by such ideal principles, but adapted to local sociocultural contexts, working within certain funding and regulatory constraints imposed on researchers. We conclude with a set of lessons learned and recommendations for the conduct of decolonial community engagement in global health research.

“ECLIPSE is different from all the projects here (*O ECLIPSE é diferente de todos os projetos que passaram por aqui*). You do not have an attitude of superiority. For the first time, we felt we were really participating. Not just giving our opinion but acting. […] When we saw the result of the videos we made with the [ECLIPSE] arts group, we felt powerful (*nos sentimos poderosas*). It was the result of our work. We realized that we were able to make that beautiful thing. We often feel tired of fighting alone, without the support of government officials. Now we feel we can count on you” (Community health worker, Brazil).

## Introduction

The global health decolonization movement is underpinned by a critical interrogation of what is regarded as “legitimate” in knowledge production (1–3). A colonialist legacy has led to a hegemonic knowledge hierarchy in which eurocentric, northern, and western perspectives are regarded as the primary legitimate knowledge base for global health research and interventions. This way of conducting global health research overlooks and undermines the values, views, and practices of the people living in the communities where the research is conducted. In this article, we argue that embedding meaningful community engagement in a global health project represents one avenue through which we can decolonize research practice and knowledge production. To illustrate this, we draw on the example of community engagement in ECLIPSE (*Empowering people with cutaneous leishmaniasis: Intervention programme to improve patient journey and reduce stigma via community education*)—an interdisciplinary applied health program based in Brazil, Ethiopia, Sri Lanka, and the UK, which aims to improve physical and mental health outcomes for people with cutaneous leishmaniasis (CL).

### Cutaneous Leishmaniasis

CL is a parasitic skin disease endemic in at least 88 countries where it presents an urgent, albeit often “neglected” public health problem (4). Transmitted by the bite of an infected sand fly, CL presents as skin lesions in one or more areas of the body. Although not fatal, CL may significantly impair quality of life (5) as visible skin lesions and disfiguring scarring may cause stigma and lead to social exclusion (5). Whilst in many cases CL lesions heal spontaneously, this may take many months and it is better for individuals to receive treatment to limit scarring and secondary bacterial infections. Biomedical treatment usually consists of a course of an anti-parasitic drug (pentavalent antimonial) which is administered *via* daily injections. The treatment may have side effects, including joint pain, muscle aches, abdominal discomfort, headache, and skin rash. More severe side effects include pancreatitis and arrhythmia (6).

CL is recognized by the World Health Organization (WHO) as a “neglected tropical disease” (NTD). It has a high prevalence in poor populations and is more common in area of conflict and in overcrowded living contexts, characterized by poor sanitation (7, 8). Like other NTDs, CL contributes to cycles of poverty and disease and presents significant risks to public health, both physical and mental, and is an impediment to socioeconomic development (9). The lack of an effective human vaccine, limited access to efficient treatment and limited local resources means that the control of CL is difficult and it remains a public health concern in affected communities.

### Research on CL

In the past, CL has received little attention and investment from research funders, partly because it is not fatal, certainly in comparison to the life threatening form of leishmaniasis, visceral leishmaniasis (VL). In recent years, though, research focused on CL has increased, partly because of a renewed attention on the control of NTDs (10). The CL research community has many sub-fields. Biomedical researchers and clinicians work toward a better understanding of the host-parasite relationship, as well as advancing drug development and other treatment protocols (11, 12). Applied health researchers are concerned with CL at an individual, community, and population level. Improving health services, public health programs and national policies are at the forefront of this strand of research (13–15). The objective of social scientists working on CL, on the other hand, is to examine how the social and cultural milieu shapes the experiences of those affected by CL (16–18).

We identified two main characteristics in the current CL literature, which influenced our own research program on CL. First, there is a tendency for CL researchers to work in disciplinary silos. Indeed, the majority of CL studies are conducted by researchers from one academic specialty and are single-disciplinary focused. For instance, research carried out with only biomedical or only applied health service researchers involved in the research teams. Second, CL researchers are often detached from the communities affected by CL and may have never engaged with community members. Often, parasitology research has been conducted by “parachute” researchers who collect data at a time and in a manner of their choosing, generally at their convenience, and exit the CL-affected communities as quickly as they appeared (19). Little communication with community members seems to take place before, during and after the study. Interaction between CL researchers and inhabitants of CL affected communities appears to be instrumental in nature—a means to an end.

The shortcomings of opportunistically “parachuting” into a community, without engaging meaningfully with affected stakeholders, are highlighted in the growing literature on community engagement, including in the global health field (19, 20). Scientific and ethical imperatives are generally cited for involving and engaging affected communities in the conduct of a research project. Robust and sincere engagement ensures that knowledge production is not dominated by academics, but that knowledge from local communities is visible and prioritized. Some argue that community engagement is particularly crucial in countries where transcontinental research, legacies of colonialism and structural inequalities may present a high risk of exploitation (21–23).

### ECLIPSE: Decolonial CL-Research

In this paper, we discuss our model of decolonial community engagement in the ECLIPSE global health research program, which aims to improve physical and mental health outcomes for people with CL. The ECLIPSE team is comprised of CL researchers from a wide range of academic disciplines (anthropology, sociology, parasitology, public health, collective health, primary care, psychology, arts, humanities, etc.) based in four countries: Brazil, Ethiopia, Sri Lanka, and the United Kingdom (UK).

ECLIPSE has four interlinked phases: (1) qualitative and ethnographic research to explore the experiences and perceptions of CL in the community, (2) quantitative research to measure CL awareness and stigma, (3) development and implementation of context-bespoke community-based CL interventions and (4) a program evaluation. Underpinning all four phases is sustained and robust collaboration with local stakeholders to guide and inform the planning and conduct of research activities in ECLIPSE.

### Objectives and Strategy of Community Engagement in ECLIPSE

From the inception of ECLIPSE, we were committed to steering away from a hierarchical and colonial way of conducting global health research, based on a fixed community engagement framework that is often dictated by researchers based in the UK. Our decolonial approach implies that the *model* for community engagement will be different in Brazil, Ethiopia and Sri Lanka. Indeed, we adopt a critical anthropological and postcolonial approach to engaging with community members.

Already from our grant application development meetings, we recognized that embedding meaningful community engagement is paramount to ensure that our research activities lead to decolonial knowledge production. For us, that means knowledge that is valued by community members. As a result, the community engagement strategy in ECLIPSE is driven by our commitment to embrace, amplify and place at the forefront community members' experiential knowledge. Therefore, we designed our community engagement in such a manner that our approach both facilitated and allowed community members to influence and direct the research in a way that is appropriate, relevant, useful, and beneficial to the communities. More specifically, four objectives inform our ECLIPSE strategy to involve and engage community members:

(1) to understand community needs and experiences around CL,
(2) to amplify the voices of community members, as well as maximize their participation and empowerment,
(3) to promote the translation of research findings into policy and practice in ways that positively impact on local communities, and
(4) to enhance cultural awareness among research team and lay foundations for future community-oriented CL and broader health research.

For this purpose, our strategy is based on the establishment of two types of groups in each ECLIPSE country: community- and policy-level groups.

**Figure 1:**
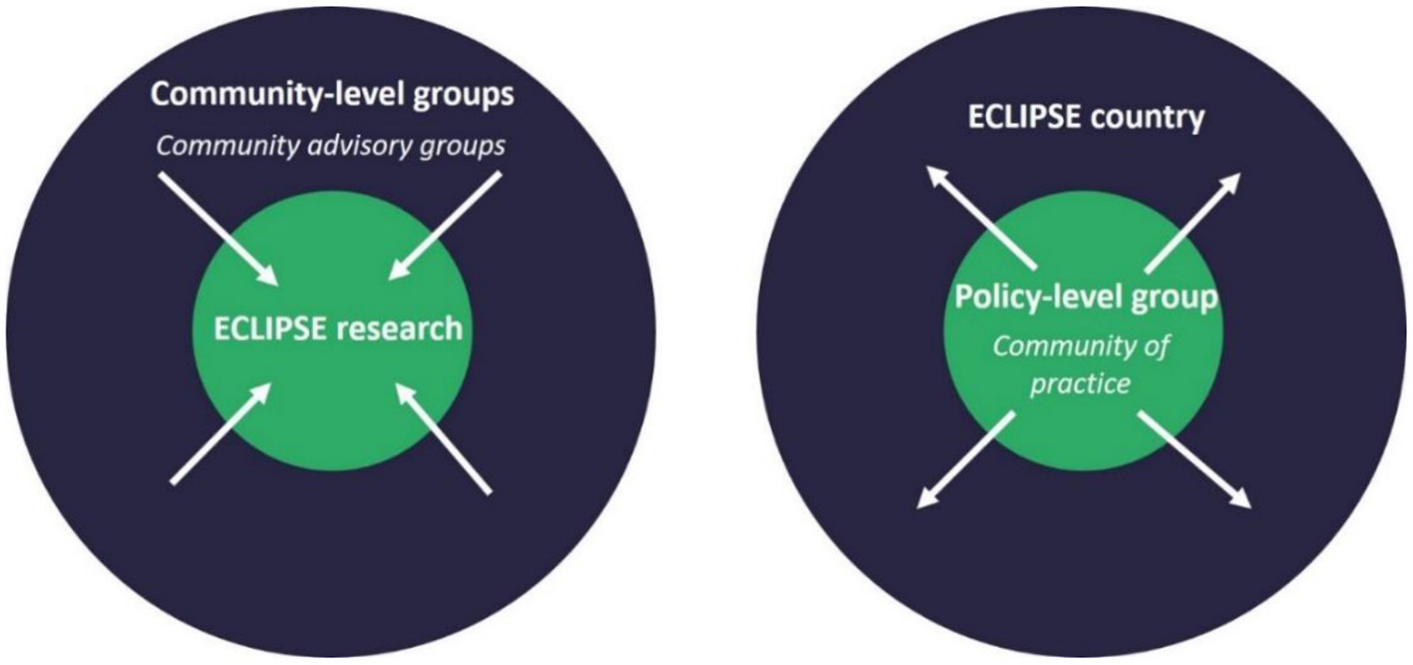
Community engagement strategy in ECLIPSE.

We established community-level groups at village/municipal level. CAGs membership is diverse and includes people with CL and individuals from their social networks, community health workers, traditional and spiritual healers, religious, and community influencers as well as other residents. Engagement *via* community-level groups is inward-facing in that it includes people who live or work in the CL-affected community and specifically focuses on how research activities are to be implemented (see Figure 1). Input and joint decision-making is sought around different aspects of the project—such as quality of public-facing material, participant recruitment processes, interpretation of findings, and how to make our activities culturally appropriate and context-bespoke.

The ECLIPSE team convenes policy-level groups at an urban-regional level. These are our Communities of Practice (CoPs). We invited stakeholders who can bring about changes in policy and practice on a regional and national level. Members include policy makers, clinicians, public health officials, religious and municipal leaders, non-governmental organizations' representatives and representatives of key sectors such as education, agriculture and health. CoP meetings are facilitated by ECLIPSE team members and have a fairly formal character given that members are invited in their professional capacity. In these ECLIPSE CoPs, members with diverse areas of expertise but who share an interest in CL are brought together (possibly for the first time) to exchange knowledge, coordinate and collaborate toward a common purpose (24, 25). In contrast to the CAGs, the focus of CoPs is outward-facing as members seek to disseminate ECLIPSE findings and interventions upwards to policymakers to promote their uptake at regional and ideally at national level (see Figure 1).

Since the format of ECLIPSE policy-level groups, in the form of CoPs, is very similar to a conventional stakeholder meeting, which is abundantly discussed in the wider literature (26–28), we focus specifically on the community-level groups, the CAGs, in this article.

## Methods

The data and material we draw on in this article were collected through qualitative research methods during the monitoring and evaluation of the community engagement practices in the ECLIPSE program.

### Monitoring and Evaluation

**Figure 2:**
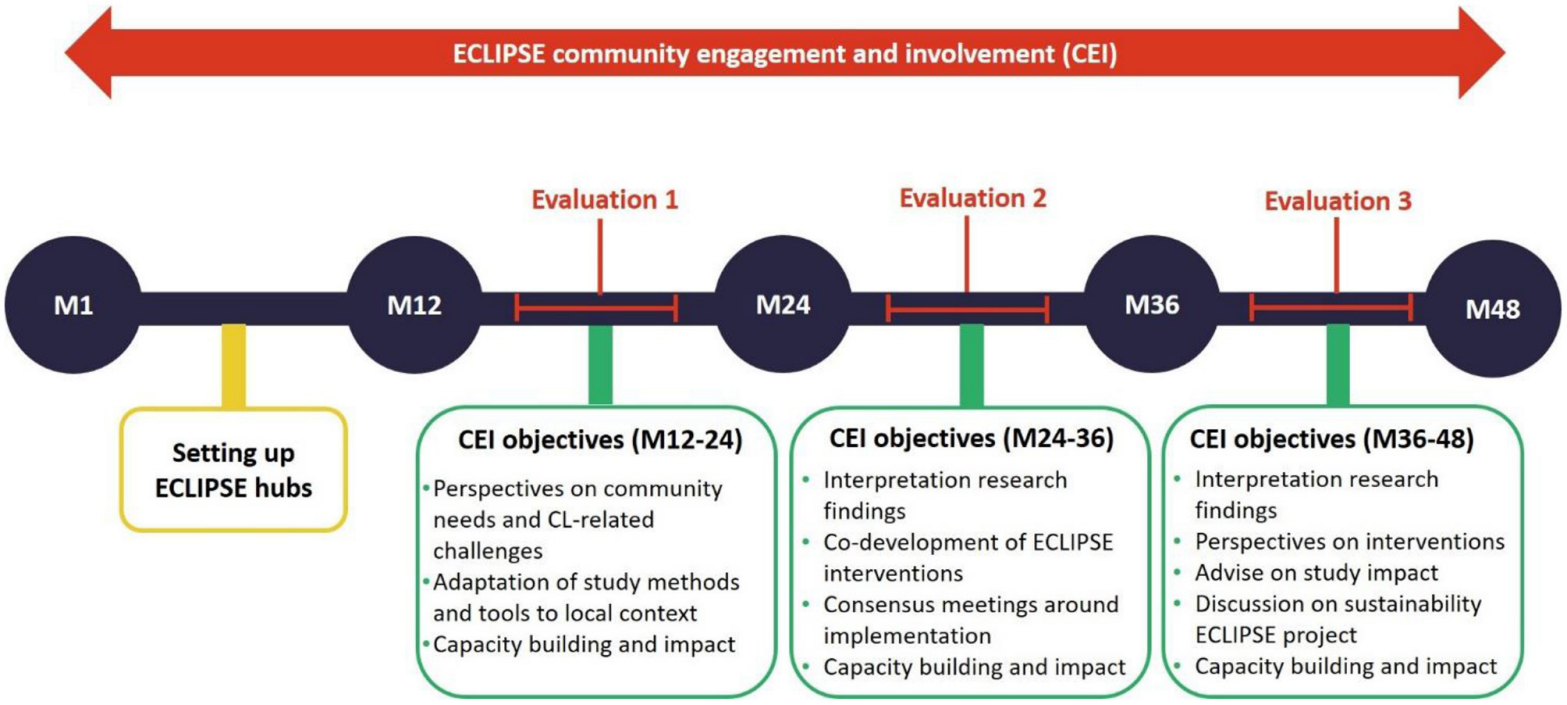
Timeline of ECLIPSE community engagement and evaluation.

At the time of writing this article, the ECLIPSE teams in Brazil, Ethiopia, and Sri Lanka have been working for up to 18 months (of a funded 48-month program). This marks our first evaluation time-point (see Figure 2).

A community engagement and involvement (CEI) team, comprised of researchers from each ECLIPSE country, is responsible for designing, implementing, and evaluating community engagement activities. This CEI team regularly meets and has detailed discussions, trainings, evaluation meetings, and co-authors progress reports. Such community engagement evaluation is paramount for ECLIPSE, as it allows us to (1) strengthen our future engagement as it progresses, (2) gather evidence about the impact of our engagement, and (3) contribute to the emerging body of literature on community engagement in global health research by disseminating best practice (29, 30). By collating and analyzing evaluation data from these three very different settings, we will be able to evaluate three different cultural models of community engagement.

### Data Collection and Analysis

We have adopted a range of qualitative research methods to continually evaluate the practice of community engagement and involvement within the cultural context of each ECLIPSE country. Data has been collected through participant observation, interviews, group discussion with CAG members, and visual methods (photo and video). We opted for such multi-method approach to evaluation as the challenges and opportunities arising from robust engagement with community members requires a sensitive approach to capture a multifaceted and nuanced picture. This might be less possible *via* simple “pre” and “post” survey-style evaluation (31).

Participant observation was undertaken by ECLIPSE team members in every meeting with community members. Researchers took detailed field notes, during and after meetings, capturing representation, atmosphere, dynamics, and details of the discussions. Some of the meetings in the community were audio-recorded, with the consent of all individuals present. Relevant parts of these recordings have been transcribed. Reflections in debrief sessions with team members about the CAG meeting were also recorded in the field notes.

A collective critical reflection—defined by Kelly et al. (32) as an opportunity “for co-learning and strengthening researchers' capacity to engage meaningfully with stakeholders”—informed our approach to data analysis. The datasets from the different country teams (containing field notes, photos, videos, and discussion transcripts) were collated, read, and analyzed by KP and LD on an ongoing and iterative basis. We applied conventional thematic analysis techniques (33, 34) to the qualitative data: cycles of coding data and discussing overarching themes identified during the coding process. Themes were reviewed until consensus was reached in the group of co-authors.

We thus adopt a critical anthropological approach to engaging with community members and it is precisely this approach we evaluate here. As anthropologists do, we critically reflect on the researcher-community members' relationship, on how research engagement and involvement is operationalized in each culturally diverse community across the three countries, and what specific changes and adaptations we made to ensure a decolonial approach to working with local residents.

### Ethical Approvals

We received approval from the ethics review committees at the four ECLIPSE institutions: from the Institute of Collective Health, Federal University of Bahia, Brazil [Ref.: 4.238.866], from the College of Health Sciences, Mekelle University, Ethiopia [Ref.: ERC/1793/2020], from the Faculty of Medicine and Allied Sciences, Rajarata University of Sri Lanka [Ref.: ERC/2020/74] and from the Faculty of Medicine and Health Sciences, Keele University, United Kingdom [Ref.: MH-200123].

All names of individuals and the names of small administrative local units within the larger regions (for instance, villages and municipalities) have been pseudonymized. We discuss ethical considerations and challenges in the sections below.

### Study Settings

**Figure 3:**
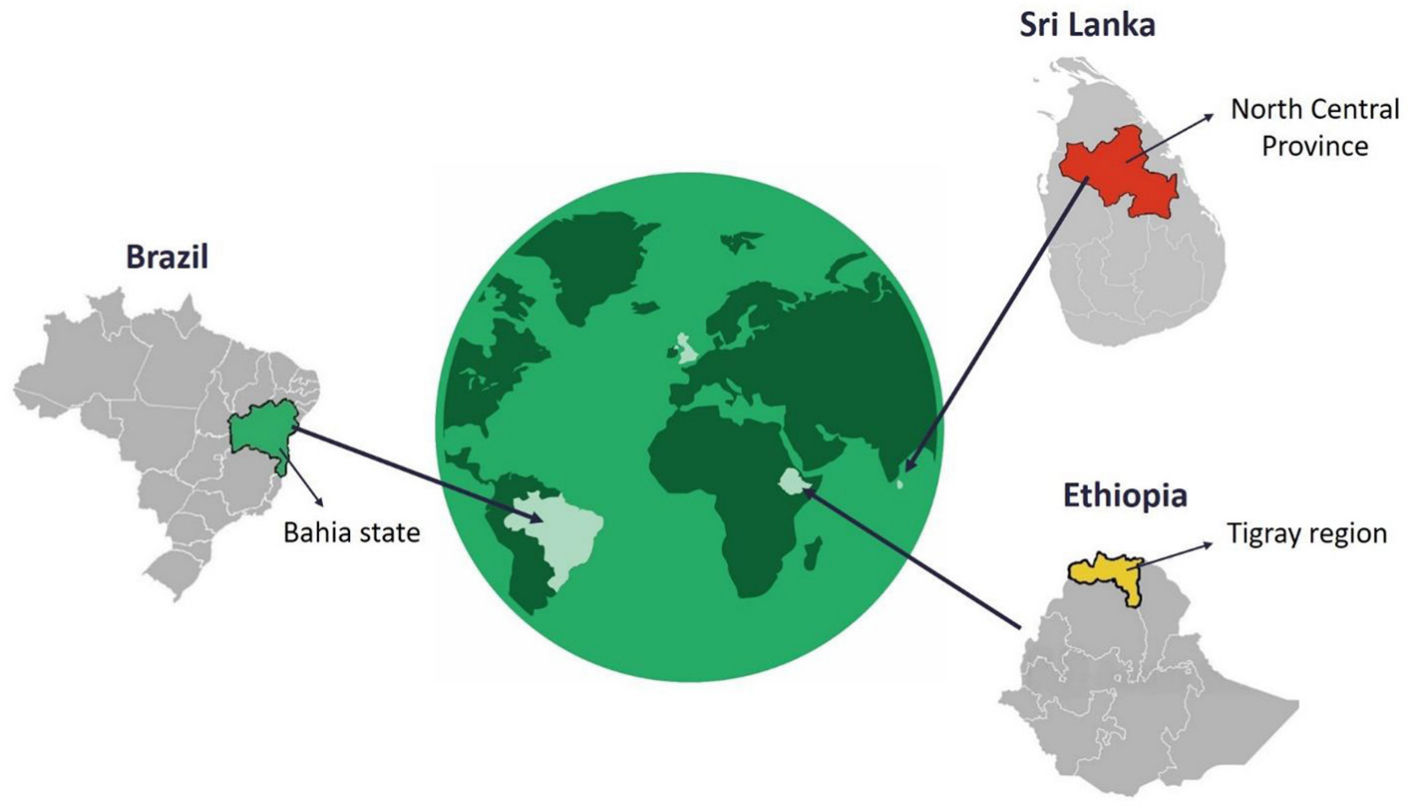
Location of ECLIPSE hubs in the three CL-endemic countries.

In the first 6 months of the ECLIPSE program, we established ECLIPSE hubs in the state of Bahia (Brazil), the Tigray region (Ethiopia), and the North Central Province (Sri Lanka; see Figure 3). Community advisory groups (CAGs) are present in each study site where ECLIPSE activities are taking place. A total of 13 CAGs have been established across the three ECLIPSE countries: Brazil (*n* = 4), Ethiopia (*n* = 6), and Sri Lanka (*n* = 3). The ECLIPSE field sites were selected on the basis of high CL prevalence. We provide here a short summary of the CL and regional context of each ECLIPSE country.

#### Bahia State in Brazil

##### CL Context

CL has been endemic in Brazil for a considerable number of years (35) and awareness of CL symptoms is relatively high in affected Brazilian communities. Mucocutaneous and disseminated CL are also found in Brazil. These are more severe forms of the disease which are more difficult to treat and can result in highly disfiguring pathology (36). Brazil is widely recognized to have one of the highest global incidence rates for CL, although most cases are found in the north of the country in the nine states of the Brazilian Legal Amazon and in the state of Bahia. The central coast of Bahia was recently identified as an intensifying hotspot for CL, with a recommendation to target this region for disease surveillance and control (36, 37).

##### ECLIPSE Hubs

Four hubs have been set up in the coastal state of Bahia, the fourth largest Brazilian state in the north-eastern part of the country (population ~ 15 million) where Portuguese is the official language. Religion in Bahia is a syncretic mix of Catholicism, Pentecostal Christianity, and the Afro-Brazilian Candomblé religious tradition (38). A rich Afro-Brazilian cultural tradition exists in Bahia, since its capital, Salvador, is the city with the largest Black population in Brazil, due to a colonial history marked by the slave trade which was concentrated in Bahian ports (39). We set up four ECLIPSE CAGs spread across three neighboring municipalities, located in primarily rural and semi-rural areas where agriculture is the main economic mode of production.

#### Tigray Region in Ethiopia

##### CL Context

CL was first reported in Ethiopia in 1913 (40, 41). While there is evidence that the disease is highly prevalent in Amhara, Tigray, and South Nations Nationalities Peoples Regional State regions of the country (40), the official numbers of CL cases appear low as disease reporting for CL (unlike visceral leishmaniasis) is not mandatory in Ethiopia (40, 42). It has been estimated that there are ~50,000 cases of CL per annum in Ethiopia (40) with a small percentage of CL cases progressing to the highly disfiguring mucocutaneous and disseminated forms as in Brazil (42). The actual burden of CL in Ethiopia is unknown: our research indicated that CL is often inaccurately recorded as a “skin infection” by healthcare professionals working at all levels—health posts, health centers, and hospitals. This aligns with what is empirically understood about the low societal awareness of CL in Ethiopia despite a potentially high, and growing, burden of disease.

##### ECLIPSE Hubs

Six ECLIPSE CAGs were established in Tigray, Ethiopia's northernmost region in the summer of 2020. The main language is Tigrinya and many Tigrayans also speak Amharic which is taught in schools. Almost 96% of the population in Tigray are Orthodox Christians. Tigrayan communities are mainly agrarian, with residents making a living from small-scale subsistence farming. Since November 2020, there has been an ongoing conflict in the Tigray region which has, in autumn of 2021, escalated to other parts of Ethiopia (43). Until time of writing (November 2021), the war was ongoing. We elaborate on this below.

#### North Central Province in Sri Lanka

##### CL Context

In contrast to Brazil and Ethiopia, CL is an emerging public health issue in Sri Lanka and there is believed to be little awareness of the condition in newly endemic regions. The first locally acquired case was reported in 1992 (44) and there has been a major increase in new cases in the north of the island over the last decade (45, 46) with a prediction of continued increases unless effective control measures are put in place (47). There is large variability in terms of reported cases across Sri Lanka, and even within districts suggesting that despite there being a mandatory requirement for healthcare professionals to report cases of CL, this is currently not always happening (48). Therefore, the national figures very likely represent an underestimation of the disease burden.

##### ECLIPSE Hubs

We established three hubs in the North Central Province (population 1.3 million), where 90% of the population is Buddhist. Local languages spoken are Sinhalese and Tamil. The three CAGs are located in rural, agricultural villages where paddy farming is the most common economic activity.

## Results

We present here findings from our analysis of data collected in Brazil, Ethiopia, and Sri Lanka, between March 2020 and September 2021, the first of three evaluation points on the community engagement strategy in the 4-year ECLIPSE program (see Figure 2). This section is structured in the four overarching themes we identified after data analysis. We illustrate each theme with examples from the ECLIPSE practice of community engagement through excerpts from field notes, ethnographic vignettes, descriptions of events, quotes from interviews, and photos.

### Establishing Community Advisory Groups

#### Brazil

We work in an area that is home to a leishmaniasis reference center. The connection between the ECLIPSE team and the healthcare professionals at that center added greater credibility to the ECLIPSE presence in the communities and boosted confidence for community members, as illustrated in the following quote:

When Alberto [ECLIPSE team member and healthcare professional at the reference center] invited me to join ECLIPSE, to be part of your group, I thought why not? Because then, when we speak to the people at the reference center, who carry out the projects together with the university in Salvador, you only bring benefits to the community and if you invite us to be part of this group, we are going to help, we are here, right? I feel very flattered and satisfied to join you because I did not expect the invitation, honestly, but here I am to help in whatever is necessary and in whatever you need (CAG member, Brazil).

#### Ethiopia

Following local customs, we employed a two-step process in the establishment of the CAGs in Ethiopia. Firstly, we sought the approval and cooperation of the local cabinets, which are composed of both government-appointed and community-elected members. Obtaining support was crucial both in symbolic terms (cabinets' endorsements legitimized ECLIPSE as a trustworthy program) and in practical terms (to facilitate the arrangement of meetings with community members in the villages). Secondly, the health extension workers, who are the most familiar with all households in one community, played a central role in informing community members about ECLIPSE and mobilizing them to attend the first introductory meetings with members of the research team.

#### Sri Lanka

We adopted both top-down and bottom-up approaches in establishing the CAGs in the North Central Province. In the top-down approach, we worked with the *Grama Niladhari*, the village officers, who have comprehensive knowledge of the families in their division. We also worked with the agricultural and public health network. These networks assimilate diverse societies such as the Farmers Society, Death and Benevolent Society and the Women's Society in each community. The bottom-up approach included several methods: transect walks through the villages, ethnographic observations, and discussions with the villagers to ask them who they would put forward as CAG members. This enabled us to ensure that community members themselves were closely involved in establishing the CAG membership.

#### Location of CAGs

The CAGs convened in very different venues, all dependent on the local infrastructure and where community members would feel at ease to engage in meaningful and, at times, difficult conversations and activities. In the low-resource settings of the Ethiopian communities in Tigray, CAG meetings often took place outside health posts or local schools as the local infrastructure was too small to convene the meetings. In Brazil, CAGs convened in venues located in the heart of these Bahian communities: in the local sports gymnasium, school and local associations' buildings. In line with the cultural context in Sri Lanka, CAGs were organized in the *Dharmashalawa* (preaching hall) of the village's Buddhist temple or in the community hall of the Funeral Society of the village.

#### Social Reality of CAGs

The material culture of the CAGs was also different in the three ECLIPSE countries. The Sri Lankan researchers, for instance, adhered to a more formal dress code and the senior female researchers wore a colorful saree to mark the special occasion of the CAG meeting. The researchers in Brazil wore project-branded T-shirts and trousers and provided community members with objects (e.g., water bottles) with the ECLIPSE logo embossed. It is important to note that the ECLIPSE logo, a bright yellow sphere overlapping with a green globe, was not appropriate to use in Brazil as this color combination is strongly associated with a specific political party. The logo was adapted to a blue-green color combination to use in Brazilian communities.

It is clear from the data that our jointly written ECLIPSE community engagement “protocol” was adapted from the start to a context-bespoke approach in establishing community groups. The different logistical requirements and cultural context in setting up CAGs were, in turn, reflected in human and financial resources ring-fenced for community engagement in each country team.

### CAG Membership and Community Representation

It is important to start this theme with highlighting that we had, and continue to have, discussions and reflection sessions on what precisely constitutes “the community” that a diverse CAG membership should reflect? In ECLIPSE, we did not identify the community solely as a geographical and place-bound entity. We employed different strategies, including purposeful invitations, snowball methods and ethnographic fieldwork, to ensure a diverse membership in each CAG. One aspect of our strategy was to seek the assistance of “influential” community figures, those with a high social status who hold symbolic power. However, it is clear from our data analysis, that each CAG (*n* = 13 in total), also includes residents who are less affluent members of already marginalized communities and who may have low visibility in the village's social structures. Here is how a Sri Lankan health official phrased it:

Let us make sure ECLIPSE is including people who are not involved in the village societies and organizations. We should give them a voice too.

#### Brazil

The constitution of the four CAGs has a certain fluidity and organicity. Most members are longtime acquaintances, neighbors, and friends, as we work in communities where everyone knows each other. Important in the Brazil CAGs is to take into account the subtle ways that gender, race, class, and age power relations play out in CAG interactions. We observed, for instance, that some members of some groups in society (men, white people, adults, those from a higher social class or members with a political position) find it easier to speak during CAG meetings. For instance, a white male political manager with a university education is more at ease to speak in comparison to a black woman who is a cook and domestic worker. We tried to minimize these subtle power dynamics to the best extent possible, by applying a range of ethical principles to our CAG meetings, including respect for diversity, recognition and appreciation of the knowledge that comes from each CAG members' lived experience. We have implemented these principles in various ways and provide some examples here. Firstly, CAG members sit in a circle (*roda*) to ensure that the seating plan does not designate any hierarchies between members. We write more about the *roda* below. Secondly, moderation of CAG meetings is important to avoid reinforcing community power structures. ECLIPSE CEI researchers do not shy away from subtly signaling those CAG members who are dominating the conversation to conclude their point, while gently encouraging more silent members to join in. Thirdly, the use of less formal language is encouraged to allow everybody, irrespective of educational levels, to participate. Finally, we also promote different communication formats, such as music, dance, and the use of images, so those who do not feel confident in speaking during meetings are able to share their experience in alternative ways. The CAGs are different in size, but have an average of 20 members, and in terms of gender balance, two CAGs have a female majority, one is gender-balanced and one group has a male majority. CAG membership includes community health workers (*Agentes Comunitários de Saúde*), members working in education or agriculture, people with CL and community leaders.

#### Ethiopia

The membership of the six CAGs established in the Tigray region was the result of a dynamic approach. After initial engagement with residents nominated by local health extension workers, a more democratic approach was viewed to be required, and hence during our next visits, the local community was invited for a meeting. Following a thorough discussion about the aims of ECLIPSE and the roles and responsibilities of CAG members, additional residents were nominated. Then, the names of those nominated by the health extension workers and those interested to join the CAGs were put up for a vote. Everybody voted for whom they wanted to represent them on their CAG. Geographic representation was deemed by community members to be a very important factor, especially for those who came from nearby hamlets (*kushet*) who wanted to ensure that a representative was elected from their locality. To address concerns regarding this, the research team decided to increase the size of the CAGs, by selecting 12 instead of 8 community members for each CAG. The final CAG membership includes community members and elders, health extension workers, people with CL and their families, local administrators, religious leaders, and traditional healers.

#### Sri Lanka

The three CAGs in Sri Lanka have a balanced composition in relation to age, gender, socio-economic position, and education background. Selection of CAG members was based on a combination of a top-down approach, where members were suggested by others (for instance, by the village officer) and a bottom-up approach, where villagers nominated themselves and other villagers. The Sri Lankan team works in the North Central Province's Anuradhapura district, known as “the cradle of Buddhism,” and where ~90% of people are Buddhists. Therefore, it was important to include Buddhist leaders as CAG members.

It is true that some members nominated by the *Grama Niladhari* are very active in common work in the village, but some are too old and some are too busy. To balance this, young people and a few women should be included (Buddhist monk).

CAG membership includes community members, religious leaders, traditional healers, people with CL and their families, teachers, local administrators, and representatives of key community groups.

### Culturally Appropriate and Context-Bespoke Engagement

All ECLIPSE researchers work within the same ethos of community engagement, which steers away from a one-size-fits-all model of CAG activities (for instance “focus groups to discuss with community members”). This theme discusses how the same set of objectives and principles have been adapted to local customs and practices by ECLIPSE researchers around the globe. For instance, all CAG members consented, in an informed way, to take part in the CAGs and for data being collected during CAG meetings. The *ways* consent was obtained and the *format* of that consent was very different across the three countries. It was tailored to the cultural context of each community, ranging from verbal consent in Ethiopia and Brazil, to written consent in Sri Lanka.

Our community engagement activities were severely affected by the COVID-19 pandemic which was declared in March 2020. We worked within radically different pandemic realities in Brazil, Ethiopia, and Sri Lanka. Some activities with Brazilian community members moved online, but such internet-based engagement was not possible in Ethiopia and Sri Lanka where community members did not have access to the internet.

#### Brazil

**Figure 4:**
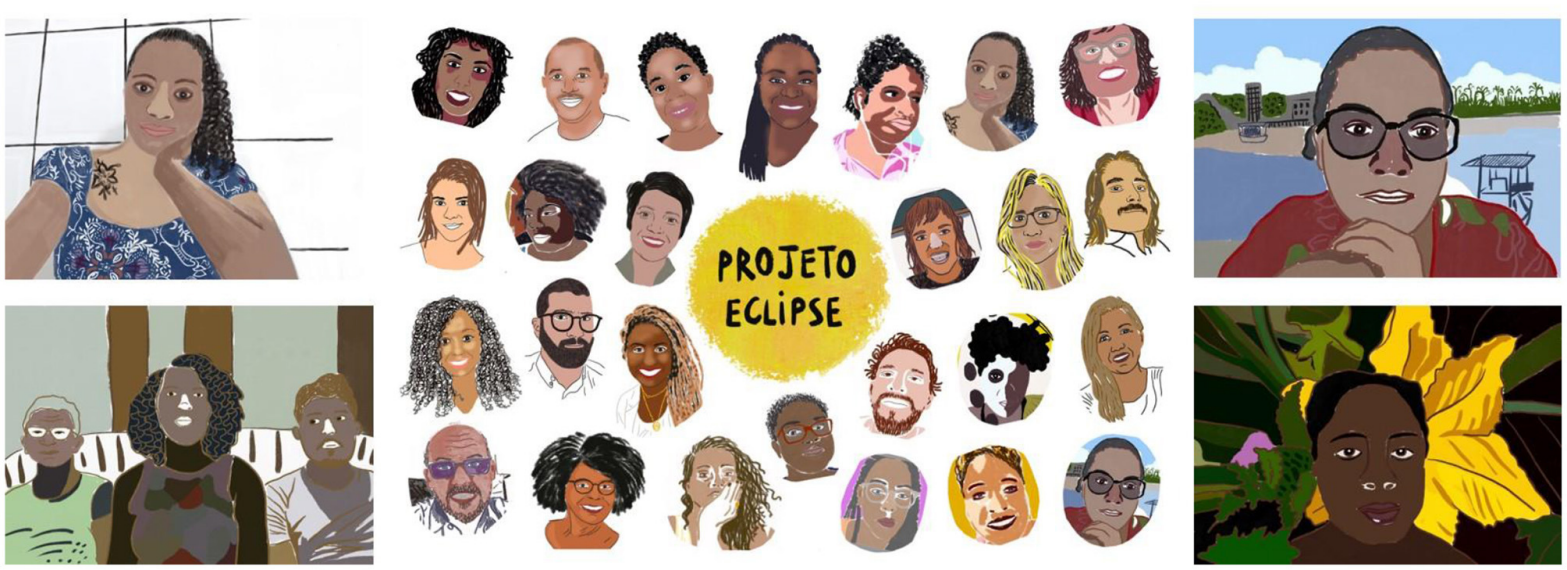
Artist and illustrator Flávia Bomfim drew faces of community members and researchers to connect with each CAG member.

The ethos of our engagement in Brazil is informed by Ubuntu principles that have roots in African philosophy and uphold the cultivation of values such as collaboration, respect, tolerance, empathy, and unity. Our plans for the initial CAG meetings in Brazil were disrupted by pandemic restrictions. Following discussion with community members, we decided to move our engagement activities online since most residents had internet access. For a few months, therefore, our engagement with CAG members was online *via* platforms like WhatsApp and Zoom. Socially engaged artists employed various artistic and creative practices to promote feelings of closeness and intimacy in such virtual meetings. These included the co-production of short videos to which both CAG members and the research team contributed by creating a short clip, filming the world just outside their window accompanied by a brief reflection. Another activity involved an artist drawing the portraits of members present, creatively addressing the challenges of doing community engagement online (see Figure 4).

**Figure 5:**
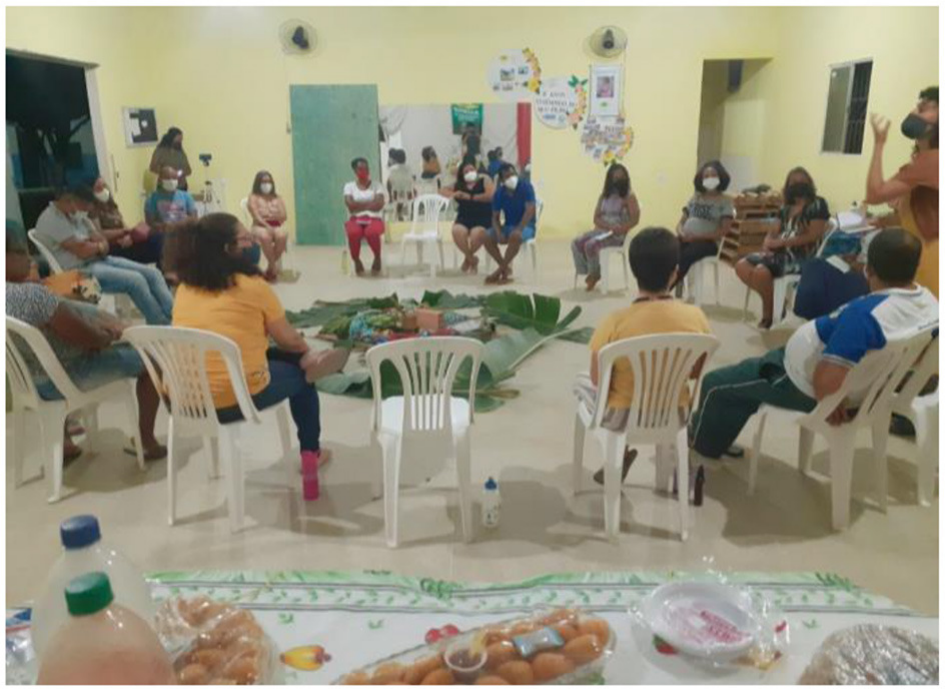
A *roda de conversa* during a Brazil CAG meeting.

**Figure 6:**
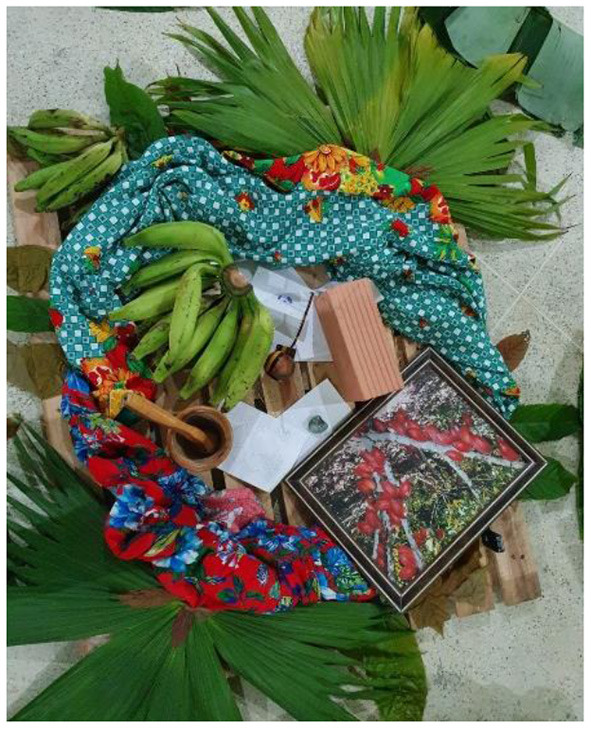
A *mandala* created by Brazil CAG members representing their community.

In person CAG meetings, which took place when public health restrictions were eased, were organized as a “talking circle” (*roda de conversa*; see Figure 5). This set-up is a popular way of organizing discussions because it facilitates an atmosphere of openness. CAG members collectively created a *mandala* during a CAG meeting, with objects brought by members which represented their community (see Figure 6). Food and other refreshments were always present during these meetings, providing an opportunity for members to socialize and connect through sharing food.

#### Sri Lanka

**Figure 7:**
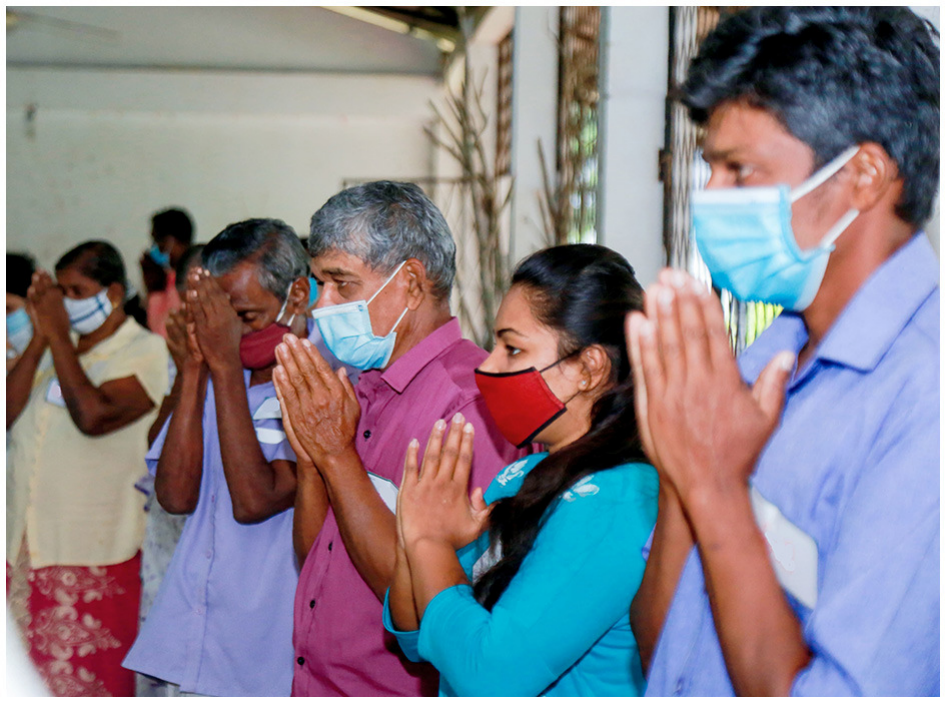
Religious observance at the start of a CAG meeting in Sri Lanka.

**Figure 8:**
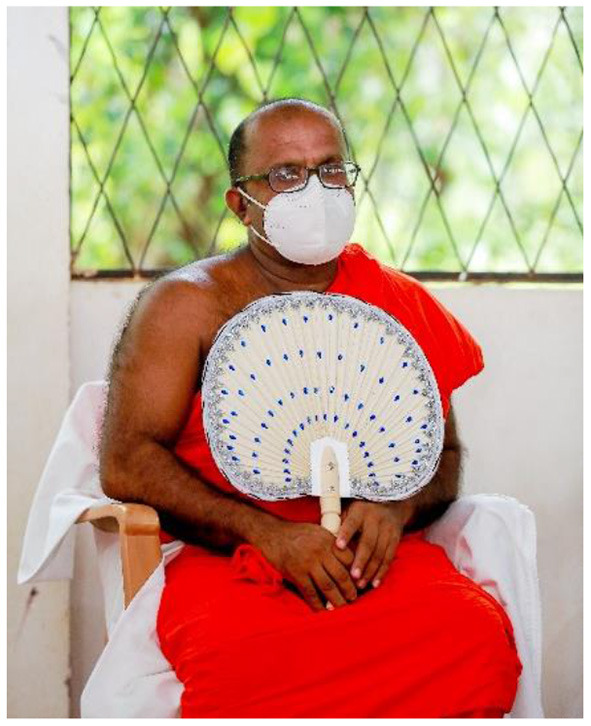
A Buddhist monk during a CAG meeting.

We were unable to engage with community members online during the pandemic, primarily due to poor internet access and low digital literacy in the villages. Sri Lankan CAG meetings take the shape of an open discussion. Following local customs, meetings informally start with refreshments and sharing food. CAG meetings commence with a Buddhist ritual of laying a white cloth on the monk's chair, which symbolize purity and is an expression of respect. Religious observances are then led by a Buddhist monk, who is a CAG member, which formally signals the start of the CAG meeting (see Figures 7, 8). Participatory methods are employed as a way to facilitate team building and collaborative knowledge production. For instance, CAG members collectively drew large maps of their villages highlighting where they seek health care and localities significant in relation to CL.

#### Ethiopia

**Figure 9:**
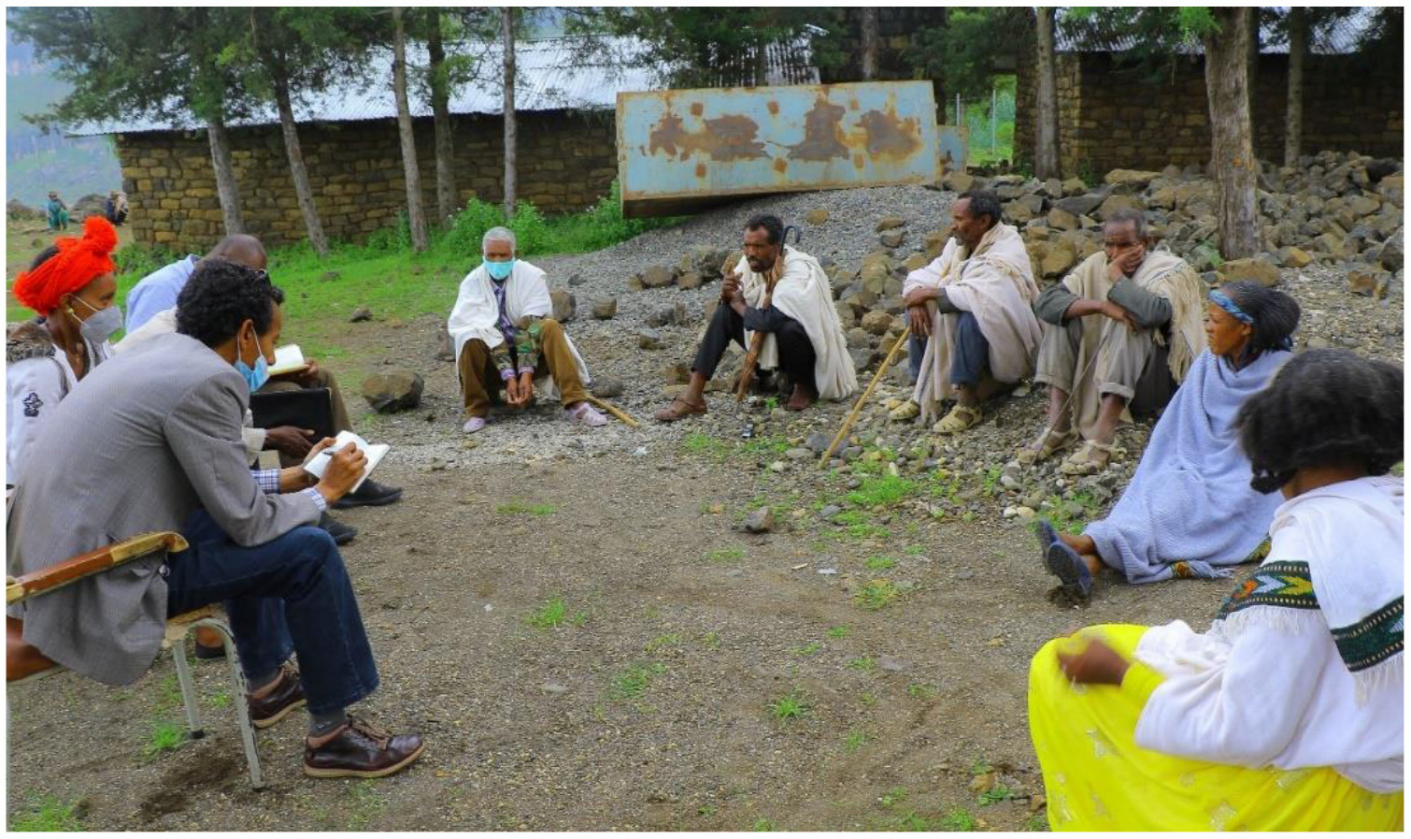
ECLIPSE team members convening a CAG meeting in Ethiopia.

It was not possible to conduct any community engagement during the pandemic lockdowns due to lack of internet access in these remote Tigrayan communities. The first CAG meetings in Ethiopia followed a conventional discussion format. We noted in these initial meetings that community members tended to offer the only available chairs to ECLIPSE team members as an expression of respect (Figure 9).

As we reflected on this during our debrief meeting, we considered how such conventions may reinforce hierarchies between the research team and community members. This, of course, posed a dilemma around following local customs (i.e., guests are offered the few chairs) to our commitment to dissolving hierarchies where possible. In Ethiopia, our engagement with community members was abruptly interrupted in November 2020 because of the outbreak of a brutal war in the Tigray region. Until the day of completing this manuscript (November 2021), we have been unable to communicate with a majority of the residents in the ECLIPSE communities because of the complete telephone and internet black-out in Tigray and the ongoing blockades and fighting, which has led to a major humanitarian crisis. In August 2021, when the heavy fighting temporarily subsided, some Ethiopian researchers were able to conduct sympathy visits to the ECLIPSE communities to express solidarity with community members.

Priorities of a community can shift rapidly when a major event or disaster happens and when residents are thus worried about basic needs such as food, shelter and physical safety. While residents in the Tigray region were interested to engage with ECLIPSE activities around CL, it is very likely that CL is not a high priority during the humanitarian crisis as a result of the ongoing war in the region. In a similar vein, as we were finalizing this paper, the state of Bahia, Brazil was hit by severe flooding. As phrased by the state governor Rui Costa, community members are “living through the worst disaster that has ever occurred” in Bahia (49). These are only two examples when ECLIPSE reevaluated the signature concerns of each stakeholder group (researchers, community members, healthcare professionals, funders, and policy makers) to adapt their CEI strategy accordingly at different time points throughout the ECLIPSE program.

### Relationships Between Researchers and Community Members

This theme revolves around the CAG members' perceptions of ECLIPSE and the evolving relationships between CAG members and researchers. From our earliest encounters with community members, we emphasized the ECLIPSE ethos of collaboration, respect, and shared decision-making. These two ad verbatim quotes, taken from early CAG meetings, illustrate how we communicate this:

This project is a little different. We usually come from the Medical Faculty and give you a questionnaire to fill. This is not one of them […]. We will be collaborating with you for a long time and we want to build this group [CAG] in this village. We are not the ones making all the decisions. It is *you* who should come together and tell us: “This is what this village needs.” This should be built on your ideas (ECLIPSE researcher, Sri Lanka).

We are not in a project that is already created. […] The great thing about the ECLIPSE project is what we as a *Grupo Consultivo Comunitário* [CAG] bring to it. This is an invitation, an invitation for all of us to build the project together (ECLIPSE researcher, Brazil).

The invitation to join an ECLIPSE CAG generated a mix of reactions among community members, both within and across the three ECLIPSE countries, ranging from skepticism on one end of the spectrum to high expectations on the other. Various factors influenced the community members' reactions. The friendly and open approach and introductions by the Brazil team, for instance, led to this positive first impression:

I was expecting suited-up people [from the research team]. When you hear “Salvador” and “university,” you think of someone with a straight posture and a [particular] way of being, a greater formality (…*a postura, o modo de ser, aquela formalidade maior*). But, what I see are people who came here and humanized us. People who reached out to us about a problem that we have, and therefore, we want to be part of finding a solution. This is the behavior that I saw from you (*Dessa necessidade de chegar nas pessoas, com aquele problema que ela tem e, assim, a gente quer fazer parte de encontrar uma solução. E esse comportamento que eu vi de vocês*). And so, for me it is very positive (CAG member, Brazil).

Community members' perceptions of the ECLIPSE research team were heavily influenced by how “universities,” “researchers,” and “research” are viewed in the community. In the Sri Lanka ECLIPSE hubs, for instance, researchers were enthusiastically received by community members, as the medical school to which they were affiliated, was held in high regard locally. This symbolic esteem tied to their identity as “researchers” thus strengthened the legitimacy of ECLIPSE. While this is a favorable starting-point, the perception that the “university” can solve the community's problems places high expectations on the ECLIPSE team, which requires our constant reflection and consideration.

In Ethiopia, we initially encountered negative attitudes from community members who had less positive experiences of being involved in past research.

We had a number of similar engagements in the past, but once the job is done nothing changes. What makes this research project different from the previous ones? (CAG member, Ethiopia).

Such concerns tied to expectations, rooted in perceived failure by other research projects/researchers to enact actual change in their communities, were particularly raised by Ethiopian and Brazilian CAG members. These perceived power imbalances led some residents to question claims around the shared decision-making. The below extract, taken from a CAG meeting in Brazil, portrays one such scenario:

***CAG member:*** The lady [ECLIPSE researcher] says [the project has not been created], but there's a plan, yes. We are arriving, we will follow this plan, we will bring important points. [but] the thing is set up, we move forward and see little points to get us somewhere. So please, do not say that project is not yet created, no.

***Co-leader project 2:*** I will give an anatomical example. The project is, at most, a skeleton. Organs, muscles, they are missing.

***CAG member:*** But it has a skeleton!

***Co-leader project 1:*** It is not a rigid skeleton (…) There is the theme, cutaneous leishmaniasis, there are some elements that are part of it: thinking about care, thinking about participation […]. To be a participatory project, the thing that is most defined in it is precisely that it needs to be carried out with the community.

This scenario clearly shows how Brazilian CAG members are active agents who critically interrogate our intentions, and most importantly, feel comfortable to challenge them during meetings. Our evaluation findings show that we should not assume that the “starting point” is a disempowered community that needs to be empowered and that this research will act as a “savior” for the community. We encountered in a number of occasions a real empowered community during CAG meetings. In summary, we are more than ever convinced that a health message to prevent and/or treat a disease should be co-created with community members rather than presented to them as a fait accompli.

## Discussion

Doing community engagement can be messy. There is no recipe book to follow, or model that can be brought to scale. It does not de-facto generate equitable health outcomes or shifts in structure inequalities. However, if it is done with an openness toward new ways of relating across differing positions of power, and new mechanisms of knowledge production with the otherwise hierarchical world global health research, it has the potential to be a positive force for change (50).

During our first period of ECLIPSE community engagement, we have debunked myths (for instance about communities being “disempowered”), critiqued our own practices (changing approaches in bringing together CAG members) and celebrated (sometimes unexpected) successes notably fruitful online engagement during a challenging pandemic context.

Our evaluation revealed a huge gap between the exemplary frameworks available in the literature and the “messy” reality of working in communities. Identifying and acknowledging that gap is a first step to avoid (re)creating a typical hegemonic model of community engagement in global health research. We have translated the ideal(istic) principles espoused by such community engagement guidance, for instance those by UNICEF and WHO, into the practical realities of “doing engagement” in low-resourced communities (51, 52). We have engaged with community members in a way that it was underpinned by idealistic principles, but adapted to local sociocultural contexts, working within certain constraints imposed on researchers. Various constraints, such as the program budget, deliverables, and milestones agreed with funders and program committees, have thus influenced the way and the degree to which these normative principles of engagement could be implemented in practice.

Colonial legacies, both in societies and global health structures, abound in the ECLIPSE countries. We recognize that the impacts of colonization are not easily reversible, but an important step to understand and change this context is to adopt the postcolonial lens (53). This is especially required in global health research underpinned by community engagement, considering the big difference that is likely to exist in terms of social status and power between researchers and community members. At the same time, we need to recognize that the colonizer exists within the colonized (54).

During the first 18 months of the ECLIPSE program, it has become abundantly clear that global health does not respect borders. The COVID-19 virus itself might not discriminate between race, class or country, but the consequences of the pandemic were certainly not the same for researchers and community members within the same country and for different ECLIPSE communities across countries. The social, cultural, and health contexts are not fixed. The brutal war in Ethiopia has brought this very starkly to the surface. The context of war and the major humanitarian crisis in Tigray will reshape the ECLIPSE activities in Ethiopia. When we are able to reconnect with the communities in this region, it is clear that we will need to rethink and redesign, together with community members, our planned engagement activities, research priorities and CL-related interventions.

### Challenging Often Taken-for-Granted Assumptions

Researcher-initiated community engagement is often seen as a gold standard in global health research (20, 22, 29, 55, 56). This perception presupposes that the exchange and relationships underpinning such engagement is reciprocal, equal, and mutually advantageous for communities and research teams. As we have shown above, we constantly critically evaluate such commonly held assumptions in our approach to community engagement. That means not only analyzing the geopolitics of power relations in global health research, but also activating and implementing the decolonial turn in community engagement in global health research. For ECLIPSE, this means critically reflecting on all too often naïve and easy assumptions that underpin many global health grant applications and global health study protocols. There are too many premises that are often taken for granted. For instance, it is assumed that power structures will not stand in the way of community engagement, researcher-community hierarchies can be dissolved, decision-making around research activities and intervention implementation will be democratic, and expectations from communities will be met. As a first step, we need to recognize the power inequities that are commonly inherent in global health programs. To state the obvious: research is funded and researchers must thus enter into a contract with their funder, an organization that expects to see deliverables and outputs. What we advocate for here, is to prioritize the other important contract, all too often overlooked or ignored, that researchers form with community members to commit to taking their perspectives seriously, maximize their participation in knowledge production, and ultimately derive better solutions for their communities' needs.

A recent, positive turn in the funding landscape of global health research, in an attempt to redress colonial legacy and roots of global research, has been the increased commitment that funding bodies attach to robust community engagement. There is now an expectation to incorporate this as a core element in global health research programs (50, 57). This has been our experience with the UK's National Institute of Health Research which funds the ECLIPSE program (58). The NIHR has indeed selected our program with its motto of “no research about us, without us” on numerous occasions as an exemplar of engaging with community members (59).

### Power Imbalances in Community Partnerships

Community engagement is fundamentally about building and sustaining relationships (31, 56, 60). Familiarity, credibility, and trust are indispensable in any research team-community relationship (61, 62). Such qualities do not appear spontaneously, but are the result of consistent, long-term (and often invisible) work on the part of researchers. In contrast to long-standing research programs (e.g., cohort studies, clinical trials) (63–65), which often benefit from a well-established presence and familiarity in the communities, it was the first time that ECLIPSE researchers were working in these specific study sites. This required us to start forging relationships with community residents as early as possible. In our case, we engaged community members *before* the program was awarded funding. We visited communities and discussed plans with local residents at the grant development stage. This was possible through a small grant from the funder. We conducted several months of preliminary work (i.e., before any actual research took place) in the form of regular visits to the study sites, informal exchanges with residents and organizing *ad-hoc* focus groups with a range of community members. Gaining the co-operation of influential bodies and individuals, to act as intermediaries (at least initially) was pivotal to arrange these encounters and attract interest. These early interactions helped to lay out the groundwork for upcoming engagement, by kick-starting a two-way familiarization process: the ECLIPSE team getting to know the context, the needs, assets and way of life of local communities, and community members getting to know the ECLIPSE researchers, the ECLIPSE vision and activities.

Nurturing trust and legitimacy among local communities in a program of this size (ECLIPSE employs 60+ individuals) requires particular considerations. In addition to the individual relationships forged between ECLIPSE researchers and community members, we worked toward ensuring that the community members endorsed the program as being meaningful for them. The establishment of the CAGs was our first step in building connections with the wider community, with the members of these groups acting as ECLIPSE ambassadors. We welcomed resistance and disagreements in CAG meetings because, when these are discussed respectfully and productively, they represent another facet of dissolving hierarchies.

Indeed, our aim in this first phase of the ECLIPSE program was to ensure that community members are aware of our commitment to take their perspectives seriously and to include them as equal partners in the knowledge production of CL. After all, we arrived as “uninvited guests” in their communities—they did not invite us. The degree to which trust was readily granted by community members was strongly influenced by contextual factors, and thus varied across the ECLIPSE countries. Mistrust in research is often deeply ingrained and sometimes a legacy of past experiences in working with researchers (66). In our case, particularly in the Brazil and Ethiopia ECLIPSE hubs, initial mistrust shown toward the project and our team members, was symptomatic of wider mistrust in institutional and governmental structures (61) which has been, in part, also reiterated by researchers—“uninvited guests”—who did not engage or engaged in problematic ways with the community, leaving behind a legacy of broken promises and unmet expectations, and thus strengthening the mistrust.

## Conclusion

**Figure 10:**
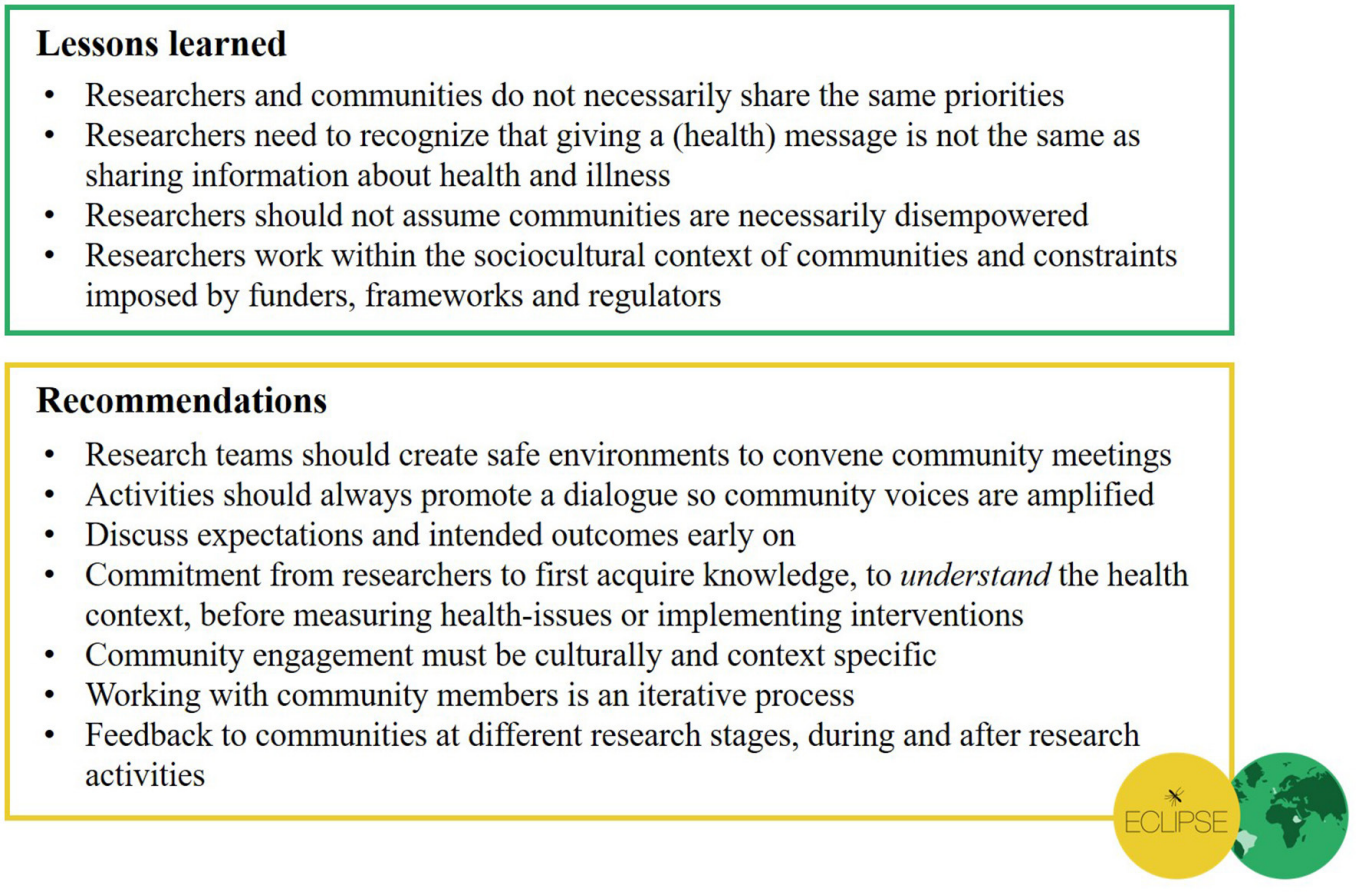
Summary of our lessons learned and recommendations for conducting community engagement in global health research.

Our experiences of practicing and discussing community engagement in global health research have led to important insights related to both theory and practice (see Figure 10). Our three main considerations after our first phase of community engagement in the ECLIPSE program are as follows. Firstly, we continue to collaborate with community members in a meaningful way, avoiding shallow tokenistic manners of engaging communities. Our overarching principle, “no research about us, without us,” is a motto decided by the community members. In implementing this principle, we aim to co-create, with the ECLIPSE communities, a safe(r), decolonial space through deconstructing dominant, western and eurocentric ways of community engagement. In doing so, we recognize that it is paramount to include the breadth of experiential knowledge of community members. Secondly, we steer away from top-down modes of engagement. If we want the ECLIPSE CAGs to be sustainable and to continue after the lifespan of the funding, it is paramount for community members to have agency and exercise control over the direction of ECLIPSE interventions and implementation. Thirdly, it is also important, during community engagement activities, to acknowledge that the ECLIPSE program will not solve all challenges and problems community members have faced for years. One theme that emerged from the data is that both researchers and community members expressed hope for change, through the planned co-production strategies.

In conclusion, drawing on our experiences to date in the ECLIPSE program, we are convinced that community engagement in global health research can be implemented in an inclusive, collaborative and equitable way, while still acknowledging the difficulties and challenges that we, and colleagues in global health research, will always face in doing so.

## Data Availability Statement

The original contributions presented in the study are included in the article. Further inquiries can be directed to the corresponding author.

## Ethics Statement

We received approval from the Ethics Review Committees at the four ECLIPSE institutions: from the Institute of Collective Health, Federal University of Bahia, Brazil [Ref.: 4.238.866], from the College of Health Sciences, Mekelle University, Ethiopia [Ref.: ERC/1793/2020], from the Faculty of Medicine and Allied Sciences, Rajarata University of Sri Lanka [Ref.: ERC/2020/74], and from the Faculty of Medicine and Health Sciences, Keele University, United Kingdom [Ref.: MH-200117]. The participants provided their informed consent to participate in this study. Informed consent was obtained from the individual(s) for the publication of any potentially identifiable images or data included in this article.

## Author Contributions

KP and LD led on writing this paper: they held regular data analysis meetings, convened meetings with co-authors to discuss findings and co-wrote the manuscript. The ECLIPSE program is co-led by LD and HP. All co-authors have read the draft manuscript, provided detailed comments and suggestions, and approved the final manuscript.

## Funding

The ECLIPSE program is funded by the National Institute for Health Research (NIHR) (NIHR200135) using UK aid from the UK Government to support global health research. The views expressed in this publication are those of the author(s) and not necessarily those of the NIHR or the Department of Health and Social Care, UK.

## Author Disclaimer

The views expressed in this article are those of the authors and not necessarily those of the NIHR or the UK Department of Health and Social Care.

## Conflict of Interest

The authors declare that the research was conducted in the absence of any commercial or financial relationships that could be construed as a potential conflict of interest.

## Publisher's Note

All claims expressed in this article are solely those of the authors and do not necessarily represent those of their affiliated organizations, or those of the publisher, the editors and the reviewers. Any product that may be evaluated in this article, or claim that may be made by its manufacturer, is not guaranteed or endorsed by the publisher.
